# Predictive value of circulating microRNA-21 levels in patients with advanced non-small cell lung cancer treated with immunotherapy

**DOI:** 10.3332/ecancer.2025.1989

**Published:** 2025-09-16

**Authors:** Cong Wang, Shan Jiang, Junfang Bi, Xu Xu

**Affiliations:** 1 Department of Pulmonary and Critical Care Medicine, Nantong Hospital Affiliated to Nanjing University of Chinese Medicine; Clinical Innovation Research Center of Nantong University; Nantong Hospital of Traditional Chinese Medicine Nantong 226000, China; 2 Medical Department, Nantong Hospital Affiliated to Nanjing University of Chinese Medicine; Clinical Innovation Research Center of Nantong University; Nantong Hospital of Traditional Chinese Medicine, Nantong 226000, China; 3 Department of Combined Traditional Chinese Medicine and West Medicine, Shijiazhuang Hospital of Traditional Chinese Medicine, Shijiazhuang 050051, Hebei, China

**Keywords:** non-small-cell lung cancer, microRNA-21, biomarker, therapeutic effect, molecular diagnostics

## Abstract

**Background:**

Pembrolizumab has been widely used to curb the disease progression of non-small-cell lung cancer (NSCLC), but 20% of patients treated with pembrolizumab have disease progression. MicroRNA-21 (miRNA-21) was highly expressed in NSCLC and promoted the occurrence of malignancy-related processes. However, the predictive value of miR-21 in NSCLC patients who underwent immunotherapy remains unknown. We aim to investigate the predictive role of miR-21 in NSCLC patients who received pembrolizumab-based combination therapy.

**Methods:**

We included 136 advanced NSCLC patients and miR-21 levels were identified. The combined positive score (CPS) was calculated and CPS≥1 was considered PD-L1 positive tumour cells in patients with NSCLC. Circulating miR-21 expressions between responders and non-responders were analysed. The overall survival (OS) and progression-free survival (PFS) according to miR-21 status were also investigated.

**Results:**

Patients categorised as responders had significantly lower expression of miRNA-21 (*p* < 0.001). Notably, miR-21 levels were also lower in patients with CPS≥1 (*p* < 0.001). According to the 50th percentile of miR-21 concentrations, patients with lower miR-21 levels had significantly improved OS (69.6 (95% CI: 63.8–75.4) versus 14.4 (95% CI: 9.9–18.9), *p*<0.001) and PFS than those with higher miR-21 levels (64.2 (95% CI: 58–70.4) versus 17.0 (95% CI: 16.5–17.5), *p*<0.001).

**Conclusion:**

MiR-21 levels were significantly correlated with the therapeutic effect and prognosis of NSCLC patients who received immunotherapy. MiR-21 holds promise as a potential biomarker of response to immunotherapy in NSCLC and it may suggest that miR-21 could be regarded as a novel indicator for prognostic prediction in NSCLC patients.

## Background

Lung cancer, which considered a highly prevalent disease worldwide, has been regarded as the leading cause of cancer-related deaths, and non-small-cell lung cancer (NSCLC) accounts for about 85% of all lung cancer cases [1]. Although new progress has been made in the treatment of NSCLC, the prognosis of NSCLC patients is far from satisfactory, with an overall survival (OS) rate of less than 20% in this population [2, 3]. Thus, improving the prognosis of NSCLC patients with new treatments is still warranted.

PD-1/PD-L1 agents, such as pembrolizumab, have been widely used as first-line therapy in advanced NSCLC [4]. A recent study by Gadgeel *et al* [5] reported the efficacy of the combination of immune checkpoint inhibitors (ICIs) and chemotherapy as a treatment option for metastatic NSCLC, and the result disclosed excellent anti-tumour effects and a safety profile for NSCLC patients. However, nearly 20% of NSCLC treated with ICIs fail to benefit from the long-term response and suffer disease progression [6, 7]. Given this, another biomarker predicting responses more accurately may be essential.

MicroRNA-21 (miRNA-21), a potential oncogene, has been reported to be involved in a variety of malignancy-related processes, such as apoptosis, cell proliferation, tumour invasion and metastasis [8]. Additionally, a previous study indicated that miR-21 is at a higher level in tumour tissues [9] and promotes the pathogenesis of lung cancer [10]. Previous studies had indicated the potential effect of miR-21 as a predictive biomarker for NSCLC; however, evidence from existing research is still insufficient and has some limitations. Moreover, the predictive value of circulating miR-21 levels in NSCLC patients who underwent immunotherapy remains largely unknown.

The present study was designed to disclose the predictive role of miR-21 in a population with NSCLC who received immune checkpoint-based treatment. We hypothesised that circulating miR-21 levels may vary between responders and non-responders (NRs) to pembrolizumab-based combination therapy and could be proposed as a marker to predict treatment response and prognosis in NSCLC patients.

## Methods

Between October 2019 and 2022, a total of 136 advanced NSCLC patients who were treated with pembrolizumab-based regimens at the Nantong Hospital of Traditional Chinese Medicine Affiliated to Nanjing University of Chinese Medicine were enrolled in this study. All patients with advanced disease received no prior systemic treatment, and the choice of chemotherapy regimen in combination with pembrolizumab was based on general standards of clinical practice. The inclusion criteria included: (I) older than 18 years old; (II) histologically diagnosed with advanced-stage or recurrent NSCLC; (III) patients who were treated with pembrolizumab-based regimens and (IV) individuals who had signed informed consents. Patients were excluded if they: (I) received <2 cycles of immunotherapy; (II) had an active second malignancy and (III) refused to participate.

Patient demographics included information on age, gender, clinical stage, Eastern Cooperative Oncology Group (ECOG) score, smoking habits and the status of brain metastasis. Previous generations of epidermal growth factor receptor tyrosine kinase inhibitors (EGFR-TKI), tumour mutational burden (TMB) and PD‑L1 status were also collected by retrospective chart review.

The research protocol complied with the Declaration of Helsinki and was recognised by the Ethics Committee and Institutional Review Board of Nantong Hospital of Traditional Chinese Medicine Affiliated to Nanjing University of Chinese Medicine. Full written informed consent was obtained in accordance with the Declaration of Helsinki from all patients before the initiation of this study.

### Quantitative assessment of miR-21

Blood samples (5 mL) of all recruited participants were collected the following morning after admission. Total RNA was extracted by exoRNeasy serum midi kit (Qiagen, Hilden, Germany). The quantitative reverse transcription-PCR (qRT-PCR) was conducted for the detection of miR-21 expression levels, which was performed on cDNA using the TaqMan^®^ MicroRNA Assay kit (Applied Biosystems, Foster City, CA, USA) according to the manufacturer’s protocol. Then qRT-PCR was performed in triplicate using an ABI Prism 7,500 sequence detection system (Applied Biosystems). The thermocycling conditions were as follows: one cycle at 95°C for 30 minutes followed by 40 cycles at 95°C for 15 seconds and 70°C for 30 seconds. The detection of miR-21 expression levels was conducted independently by the same laboratory technician who was blinded to the clinical status and outcome of included patients, which may minimise the technological fluctuations and batch effects of detection of miR-21.

### Statistical analysis

For statistical analysis, all participants were categorised as either responders (R; defined as best radiographic response of either complete response, partial response or stable disease or (NR; defined as radiographically progressive disease). Response of treatment was assessed using a variety of imaging modalities every 8 weeks for 6 months. PD-L1 expression was detected in samples from endoscopic biopsy before therapy using the PD-L1 VENTANA assay based on the manufacturer’s protocol. The combined positive score (CPS) was calculated based on PD-L1 stained slides. CPS≥1 was considered PD-L1 positive tumour cells in patients with NSCLC. TMB was defined as the number of non-synonymous mutations per megabase.

In this study, we need to have 95% confidence and 80% power to detect a difference of 0.07 from the presumed value of AUC = 0.7. Therefore, the required sample size of this study is 114. The Mann–Whitney *U* test was applied for comparison of differences in the miR-21 expression between groups. Spearman's rank correlation analysis was performed to explore the association between miR-21 levels and the TMB. The progression-free survival (PFS) and OS were calculated by the Kaplan–Meier method and survival estimates were compared using the log-rank test. The cumulative incidence of OS and PFS was presented and risk factors were evaluated by Cox regression. The area under the receiver operating characteristic curve (AUC) was also computed to assess the potential of miR-21 as a biomarker for pembrolizumab response. All differences were considered significant at* p* < 0.05. Data were subjected to statistical analysis using SPSS 24.0 (SPSS Software Inc., Chicago, IL, USA) and GraphPad Prism 8.02 software (GraphPad Software Inc., La Jolla, CA, USA).

## Results

Between October 2019 and 2022, 136 consecutive patients with advanced NSCLC received pembrolizumab-based regimens and were included in the main cohort. The median age was 56 (41–68) years old and 86 (63.2%) were male. PD-L1 CPS was available in all included patients before treatment, and 58 of 136 patients (42.6%) were PD-L1 positive (CPS≥1). The median TMB was 11 mutations per megabase (mut per MB; interquartile range: 6–15). 86 patients (63.2%) were current or former smokers and 25 (18.4%) reported metastatic disease at enrollment. The baseline characteristics of these patients were listed in Table 1.

Among all the 136 patients, 61 survived at the end of follow-up and the overall mortality was 55.1%. All included patients were dichotomised into low and high groups according to the 50th percentile of miR-21 concentrations in the overall distribution. The number of patients with EGFR-mutation was significantly increased in microRNA-21 levels above the median (*p* = 0.017). The mortality in the group with miR-21 level below the median was 27.9%, while the mortality in the group with miR-21 level above the median was 83.8%.

**Figure 1.**
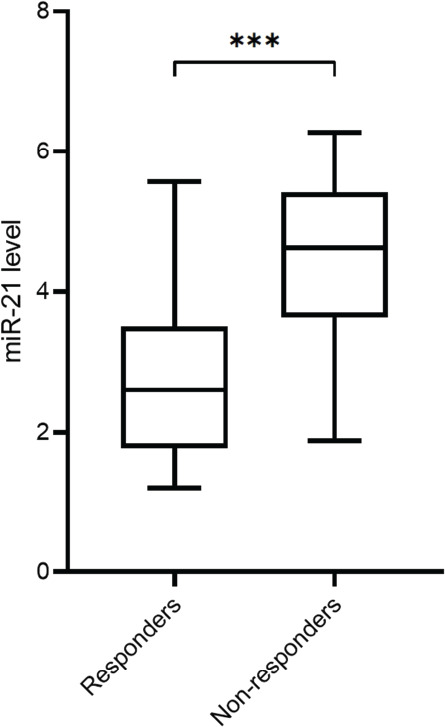
(a): The relative expression of circulating miR-21 between the responders and NR in NSCLC patients. (b): The relative expression of circulating miR-21 between NSCLC patients with CPS≥1 or CPS<1.

**Figure 2.**
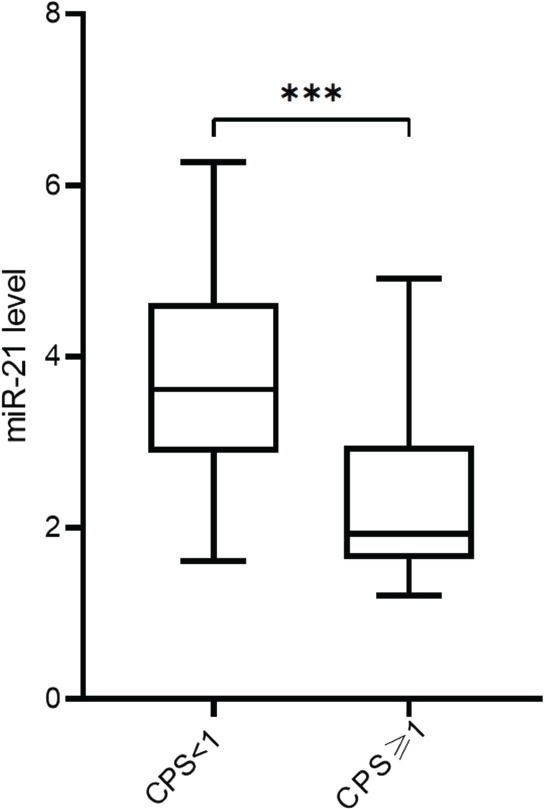
The Spearman correlation between miR-21 level and TMB.

Based on the analysis of circulating miR-21 expression between responders and NR, significantly lower expression of miR-21 (*p* < 0.001) was observed in patients categorised as responders (Figure 1a). Furthermore, circulating miRNA-21 levels were also significantly lower in patients with CPS≥1 (*p* < 0.001, Figure 1b) in comparison to patients with CPS<1. Figure 2 shows the correlation analysis between TMB and miR-21 content, and the TMB level was negatively associated with miR-21 levels (*r* = −0.68, *p* < 0.001).

**Figure 3.**
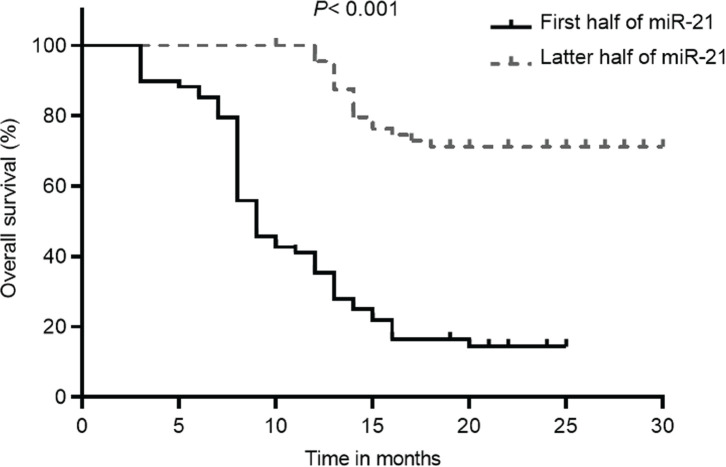
OS of patients with NSCLC with different levels of miR-21 according to the 50th percentile of miR-21 concentrations in the overall distribution.

**Figure 4.**
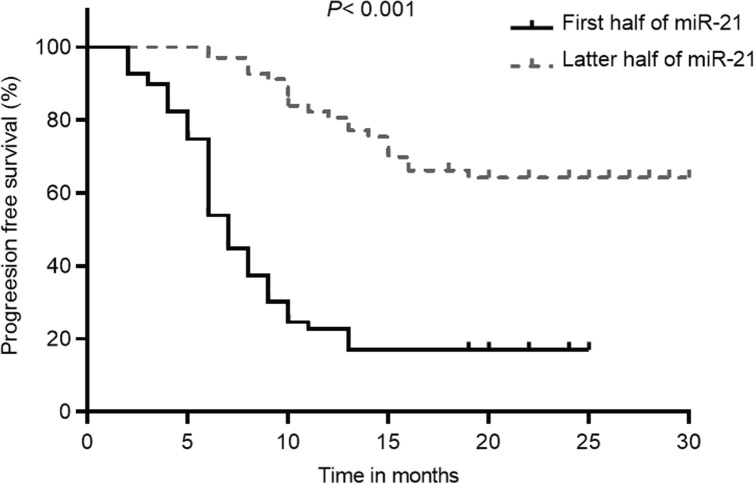
PFS of patients with NSCLC with different levels of miR-21 according to the 50th percentile of miR-21 concentrations in the overall distribution.

Kaplan–Meier analysis showed that, according to Figure 3, patients with lower miR-21 levels had a significantly improved OS than those with higher miR-21 levels (69.6 (95% CI: 63.8–75.4) versus 14.4 (95% CI: 9.9–18.9), *p* < 0.001). Additionally, patients in the low miR-21 group also had significantly higher PFS compared to those with high miR-21 levels (64.2 (95% CI: 58–70.4) versus 17.0 (95% CI: 16.5–17.5), *p* < 0.001) (Figure 4). The initial statistical analysis of the univariable Cox regression analysis indicated that miR-21 level was associated with the OS (HR: 2.32, 1.88–2.86, *p* < 0.001) and PFS (HR: 2.19, 1.79–2.68, *p* < 0.001). After adjustments for age, smoking, histology type, ECOG status and PD-L1, miR-21 level remained a significant determinant for the OS (HR: 1.78, 1.39–2.29, *p* < 0.001) and PFS (HR: 1.66, 1.29–2.13, *p* < 0.001).

AUC analysis was conducted to examine the potential of miR-21 as a biomarker in differentiating pembrolizumab responders, and the AUC was 0.876 (95% CI, 0.807–0.944). Then, we assessed the diagnostic accuracy of miR-21 on pembrolizumab response in identifying lung cancer patients; the clinically actionable threshold of miR-21 was 3.71 and the estimated sensitivity and specificity were 75.0% and 86.5%.

**Figure 5.**
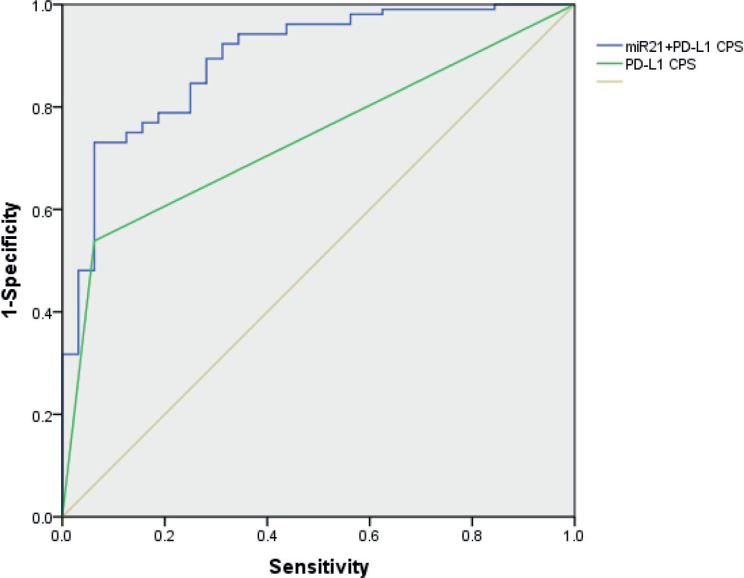
The ROC curve of miR-21 level and PD-L1 CPS for the ability to differentiate the responders of pembrolizumab in NSCLC patients.

Figure 5 illustrates that the AUC was 0.738 (95% CI, 0.651–0.825) of PD-L1 CPS, while the additional value of miR-21 on top of PD-L1 CPS showed that the AUC of the combined biomarker approach is 0.893 (95% CI, 0.831–0.956). Thus, miR-21 adds value beyond PD-L1 in predicting the response of pembrolizumab in NSCLC patients.

## Discussion

In this study, we observed that miR-21 affected the prediction of the prognosis of NSCLC patients who received pembrolizumab-based combination therapy. The present research may disclose how useful miR-21 expression is in contributing to NSCLC patients’ monitoring. In this study, the level of miR-21 was found to be significantly linked to the therapeutic effect in NSCLC patients. Additionally, those with higher level miR-21 showed poorer prognosis than patients with lower levels, and it may indicate that miR-21 could be a novel indicator for prognostic prediction in NSCLC patients.

The pathogenesis of lung cancer involves a large number of biological processes, which are connected with many key genes. Currently, there are many means for diagnosing lung cancer at an early stage by screening high-risk patients, such as smokers, to improve the prognosis [11]. Liquid biopsy is advantageous because it is easy to obtain and allows for serial monitoring [12]. Among them, microRNAs could be considered as a well-known marker of carcinogenesis and a predictor of patients. Previous studies indicated that abnormal circulating miRNAs levels were helpful for predicting the prognosis of lung cancer [13].

Among the many microRNAs that have been correlated to the pathogenesis of cancer, miR-21 was identified as an oncogenic miRNA. It affects cancer progression, including influence on proliferation, apoptosis, stemness and chemo-resistance of cancer cells [14]. Previous research reported that overexpression of miR-21 is related to lung cancer [15]. A previous study shows that the up-regulation of miR-21 is significantly associated with lung cancer induced by hypoxia [16]. Current evidence demonstrates that miR-21 was upregulated in NSCLC patients carrying p53 mutation and the patients with increased miR-21 expression had a poorer prognosis [17]. In previous studies, miR-21 was found to be inversely correlated with the expression of PTEN and PDCD4 and positively correlated with the PI3K/Akt pathway [18, 19]. Additionally, EGFR has been considered to influence the maturation of microRNAs, and the vital connection between the EGFR mutation and circulating miR-21 expression level has been reported by Shen *et al* [20]. Moreover, NSCLC patients with EGFR mutation had considerably elevated miR-21 levels [21]. In view of this, the upstream regulators (hypoxia, EGFR signaling and cytokines) and downstream targets (PTEN, PDCD4 and STAT3) of miR-21 may mediate the response of pembrolizumab.

MiR-21 expression is high in cancer tissues, and circulating miR-21 is readily detectable in blood samples, making it a valuable biomarker in association with the susceptibility to some drugs. In NSCLC patients, miR-21 can silence drug resistance and reduce phosphorylation of Akt to modulate the transcriptional factor E2F-1 and Twist expression levels. Thus, miR-21 may play a vital role in drug resistance [22]. Increased miR-21 expression may confer resistance to multiple drugs through hypoxia indirectly, which facilitates the development of drug resistance and induces numerous factors that impact tumourigenesis [18]. Additionally, miR-21 levels impact immune response, T-cell infiltration and PD-L1 expression. Additionally, miR-21 levels impact immune response, T-cell infiltration and PD-L1 expression. PD-L1 expression was increased in miR-21-deficient macrophages, which inhibit phagocytic anti-tumour immunity. Tumour cells stimulate the miR-21 expression in macrophages and suppress the activation of STAT1 and NF-κB by inhibiting STAT1 and JAK2 expression, and prevent anti-tumoural M1 polarisation [23]. In addition, research also reported that miR‐21 links to pro‐tumour immune responses with elevated M2 macrophages, which suggests that higher levels of miR‐21 may induce drug resistance against immunotherapy [23, 24]. Additionally, flow cytometry analysis revealed that inhibition of oncogenic miR-21 by P21 confers immunogenicity by enhancing CD8+ T cell infiltration in tumour tissues [25]. However, these studies have not reported the predictive and prognostic role of miR-21 in NSCLC patients who underwent immunotherapy.

Immunotherapy may have a limited response rate and may cause drug-related adverse events in some patients. Hence, further understanding of indicators in predicting treatment response is necessary. To date, there have been no widely established biomarkers in clinical practice for monitoring treatment response in NSCLC. Moreover, the evidence about specific connections between microRNAs expression level and efficacy, together with the prognosis of NSCLC patients who underwent immunotherapy, is still scant. Thus, a study is warranted to disclose the predictive value of miR-21 in reflecting the efficacy and prognosis of the NSCLC population receiving pembrolizumab-based combination therapy.

Immune activation within the immunosuppressive tumour microenvironment, resulting from reduced miR-21 expression, enhances sensitivity to immune checkpoint blockade therapy. The result of this study translates into clinically meaningful survival benefits in patients with a lower level of miR-21 when receiving pembrolizumab, while patients with higher miR-21 levels predict poor pembrolizumab response, and these patients should receive pembrolizumab and chemotherapy. In view of the elevated miR-21 levels were closely linked to adverse outcomes for individuals with NSCLC. As an oncogenic miRNA, miR-21 targets PTEN, resulting in uncontrolled activation of PI3K/AKT, fostering cancerous cell survival, proliferation and evasion of apoptosis. Thus, anti-miR-21 therapies may hold therapeutic promise in NSCLC patients, and further research is imperative for a comprehensive understanding of the effects of these therapeutic strategies.

As for the potential role of miR-21, we believe that the lower levels of miR-21 were connected with improved clinical outcomes in NSCLC patients who underwent immunotherapy. A major limitation was that the follow-up period of this study was relatively short and the follow-up of the included patients in this study is ongoing in our center, which will permit us to validate our data. Second, the small number of participants available for analysis might have underpowered the planned analysis and the study’s single-center design may limit the generalisability of the findings. However, the results suggest that the choice of miR-21 in a population with NSCLC who received pembrolizumab-based combination therapy. As a pilot study, the results should be confirmed by larger multicenter studies to validate the findings and expand the available clinical data. Third, the predictive role of miR-21 needs to be prospectively investigated to determine its reliability. Fourth, longitudinal monitoring of miR-21 is missing in this study, although the serial monitoring of miR-21’s level gave a dynamic change of this marker. Last but not least, due to limitations of laboratory conditions, it is impractical to conduct the related experiments at present. Additional studies to investigate the correlation of miR-21 levels with immunosuppressive features, such as immune response, T-cell infiltration and M2 macrophage presence, would be beneficial to our study. Certain experiments in the future will be warranted to supplement the findings of this study.

## Conclusion

Taken together, the present study investigated the predictive role of miR-21 in a population with NSCLC who received pembrolizumab-based combination therapy, and we found that the circulating miR-21 expression levels were significantly connected with the therapeutic effect of NSCLC patients. In addition, we also investigated the value of miR-21 for prognostic prediction in NSCLC patients. However, this study was conducted in a single center and with a limited sample size, further study might be needed in the future.

## Conflicts of interest

The authors have nothing to disclose.

## Author contributions

Conception and design: CW, XX.

Development of methodology: SJ, JFB.

Acquisition of data: SJ, JFB.

Writing, review and/or revision of the manuscript: CW, XX.

## Data availability statement

The data that support the findings of this study are available on reasonable request from the corresponding author.

## Figures and Tables

**Table 1.** Clinical and demographic characteristics of included NSCLC patients.

Characteristics	microRNA-21		*p* value
	Below the median	Above the median	
Age, median (range), year	55 (41–66)	57 (41–68)	0.610
Men, No. (%)	39 (57.4)	47 (69.1)	0.155
Smoker or former smoker	32 (47.1)	43 (63.2)	0.058
Clinical stage			
IIIB	12 (17.6)	7 (10.3)	0.463
IV	49 (72.1)	53 (77.9)	
Recurrence	7 (10.3)	8 (11.8)	
Previous EGFR-TKI			
No TKI	46 (67.7)	31 (45.6)	0.017
1st Generation	10 (14.7)	23 (33.8)	
2nd Generation	12 (17.6)	14 (20.6)	
Histological subtype, No. (%)			
Adenocarcinoma	32 (47.0)	29 (42.6)	0.663
Squamous	27 (39.7)	32 (47.1)	
Other	9 (13.2)	7 (10.3)	
Brain metastasis			
Yes	9 (13.2)	16 (23.5)	0.121
No	59 (86.8)	52 (76.5)	
ECOG performance status			
0	30 (44.1)	35 (51.5)	0.39145
1	38 (55.9)	33 (48.5)	
PD-L1 CPS≥1			
Yes	45 (66.2)	13 (19.1)	<0.001
No	23 (33.8)	55 (80.9)	

Abbreviation: NSCLC: Non-small-cell lung cancer; EGFR-TKI: Epidermal growth factor receptor tyrosine kinases inhibitor; CPS: combined positive score
